# The Spread of Influenza A(H1N1)pdm09 in Victorian School Children in 2009: Implications for Revised Pandemic Planning

**DOI:** 10.1371/journal.pone.0057265

**Published:** 2013-02-26

**Authors:** James E. Fielding, Isabel Bergeri, Nasra Higgins, Heath A. Kelly, Julian Meagher, Emma S. McBryde, Rodney Moran, Margaret E. Hellard, Rosemary A. Lester

**Affiliations:** 1 Victorian Infectious Diseases Reference Laboratory, Melbourne, Victoria, Australia; 2 Victorian Government Department of Health, Melbourne, Victoria, Australia; 3 National Centre for Epidemiology and Population Health, The Australian National University, Canberra, Australian Capital Territory, Australia; 4 Burnet Institute, Melbourne, Victoria, Australia; 5 Victorian Infectious Diseases Service, Royal Melbourne Hospital, Melbourne, Victoria, Australia; 6 Department of Medicine, The University of Melbourne, Melbourne, Victoria, Australia; 7 Department of Epidemiology and Preventive Medicine, Monash University, Melbourne, Victoria, Australia; University of Hong Kong, Hong Kong

## Abstract

**Background:**

Victoria was the first state in Australia to experience community transmission of influenza A(H1N1)pdm09. We undertook a descriptive epidemiological analysis of the first 1,000 notified cases to describe the epidemic associated with school children and explore implications for school closure and antiviral distribution policy in revised pandemic plans.

**Methods:**

Records of the first 1,000 laboratory-confirmed cases of influenza A(H1N1)pdm09 notified to the Victorian Government Department of Health between 20 May and 5 June 2009 were extracted from the state’s notifiable infectious diseases database. Descriptive analyses were conducted on case demographics, symptoms, case treatment, prophylaxis of contacts and distribution of cases in schools.

**Results:**

Two-thirds of the first 1,000 cases were school-aged (5–17 years) with cases in 203 schools, particularly along the north and western peripheries of the metropolitan area. Cases in one school accounted for nearly 8% of all cases but the school was not closed until nine days after symptom onset of the first identified case. Amongst all cases, cough (85%) was the most commonly reported symptom followed by fever (68%) although this was significantly higher in primary school children (76%). The risk of hospitalisation was 2%. The median time between illness onset and notification of laboratory confirmation was four days, with only 10% of cases notified within two days of onset and thus eligible for oseltamivir treatment. Nearly 6,000 contacts were followed up for prophylaxis.

**Conclusions:**

With a generally mild clinical course and widespread transmission before its detection, limited and short-term school closures appeared to have minimal impact on influenza A(H1N1)pdm09 transmission. Antiviral treatment could rarely be delivered to cases within 48 hours of symptom onset. These scenarios and lessons learned from them need to be incorporated into revisions of pandemic plans.

## Introduction

Influenza A(H1N1)pdm09 was identified in Mexico and the United States (US) in April 2009 [1]. It spread rapidly around the globe and by 12 May cases had been reported in 30 countries, including Australia’s first case in the state of Queensland on 9 May [2], [3]. The second Australian case was reported in Victoria eleven days later [4], after which notifications of confirmed cases in Victoria accelerated much more rapidly than in other states and territories [5]. The vast majority of these cases occurred in metropolitan area of the state capital Melbourne. By early June there were over 1,000 cases in Victoria [6], more than all the other Australian states combined. This lead to Melbourne being referred to in some popular media outlets as the “swine flu capital of the world” [7].

Australia’s response to influenza A(H1N1)pdm09 was undertaken in accordance with the phases described in the Australian Health Management Plan for Pandemic Influenza (AHMPPI) [8], which was shifted from *Delay* to *Contain* on 22 May in response to evidence of local transmission in Victoria [3]. During the *Delay* and *Contain* phases testing was recommended for all suspected cases in the community. As the number of notified cases in Victoria increased, investigation of all suspected cases became unsustainable and Victoria announced its move to a *Modified Sustain* phase on 3 June; other jurisdictions remained in *Contain* [4]. Following an announcement by the Australian Government on 17 June, all Australian jurisdictions subsequently moved to a new *Protect* phase [3], with Victoria implementing this phase on 23 June. Testing during *Modified Sustain* and *Protect* was generally focussed on those most at risk of moderate to severe illness (including those with certain chronic medical conditions or obesity, Indigenous Australians, pregnant women, young children and infants and health care workers) and those presenting with moderate to severe disease [3], [4].

School closure and distribution of antiviral medication are important components of the recommended response to pandemic influenza and both strategies were implemented in Victoria [9]. We reviewed the epidemiological data of the first 1,000 notified cases of confirmed influenza A(H1N1)pdm09 in Victoria to gain further insights into viral transmission among school children and the implications of this transmission on administration of oseltamivir for treatment and prophylaxis and for school closures. Insights from this study can inform revised pandemic plans.

## Methods

Laboratory confirmed influenza is a scheduled Group B notifiable disease under the Victorian Health (Infectious Diseases) Regulations 2001. Medical practitioners and pathology services are required to notify cases, including prescribed demographic, illness and outcome fields, to the Victorian Government Department of Health (the department) in writing within five days of diagnosis.

All confirmed influenza A(H1N1)pdm09 cases notified during the *Delay* and *Contain* phases were investigated and demographic and illness data were collected. Data on school attended were also collected for cases aged from five to 17 years inclusive. Attempts were made to identify all close contacts of confirmed cases – defined as within one metre of the confirmed case (while infectious) for more than 15 minutes or in the same room as a confirmed case for more than four hours – for provision of prophylaxis and/or quarantine advice as indicated.

During the *Delay* and *Contain* phases, testing for influenza A(H1N1)pdm09 at the state reference laboratory was authorised by the department for all suspected cases, defined as a person with fever and recent onset of at least one of rhinorrhoea, nasal congestion, sore throat or cough. A case was confirmed if influenza A(H1N1)pdm09 was detected by polymerase chain reaction.

All case data were entered into the department’s Notifiable Infectious Diseases Surveillance (NIDS) database. Records of the first 1,000 notified cases of confirmed influenza A(H1N1)pdm09 cases were extracted from the NIDS database and analysed descriptively with Microsoft Excel software. Using Stata (Version 10.0) statistical software, the χ^2^ and Fisher’s exact tests were used to compare proportions, and the Mann-Whitney U test to compare time between diagnostic events and the number of contacts per case. A p value of less than 0.05 was considered significant. Mapping was undertaken with ArcGIS software.

### Ethics Statement

Approval from the Victorian Government Department of Health Human Research Ethics Committee was not required for this study because data were collected as part of regulated notifiable disease surveillance. Influenza (laboratory confirmed) is a scheduled notifiable disease in Victoria and notification of all cases and prescribed data fields to the Department of Health is mandatory under the Health (Infectious Diseases) Regulations 2001. Written consent from patients is not required for notification of a notifiable infectious disease. Data in the study were used and reported within the requirements of the Victorian Health Records Act 2001.

## Results

**Figure 1:**
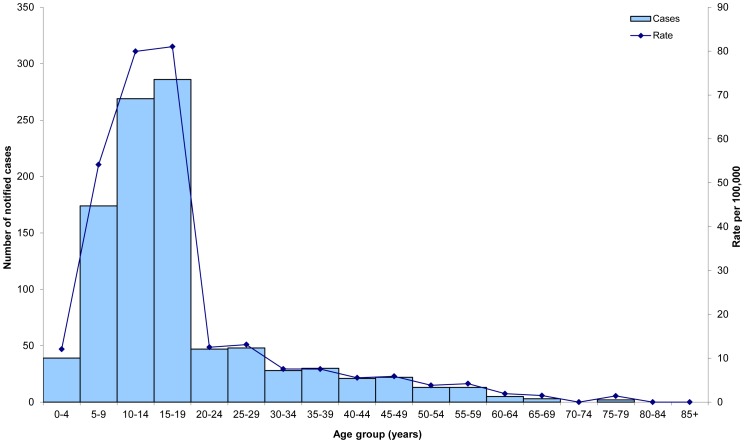
Confirmed influenza A(H1N1)pdm09 cases and rate per 100,000 population by age group, Victoria, 2009.

The initial detection of influenza A(H1N1)pdm09 in Victoria has been described in detail elsewhere [10], [11]. Briefly, the first case was confirmed on 20 May and increased to a peak of more than 250 cases on 2 June; the 1,000th case was confirmed on 5 June. Only eight (0.8%) of the first 1,000 notified cases had a reported history of travel to an area affected by influenza A(H1N1)pdm09. Ages of cases ranged from five months to 79 years with a median of 15 years. The modal five-year age groups were 10–14 and 15–19 years (figure 1).

Unspecified symptoms were reported for 14 cases and “flu-like symptoms” reported for 25 cases. An illness onset date was nominated for 389 cases but no symptoms were reported. Data about specific symptoms were available for 520 cases (52%) and are shown in table 1. Cough was the most commonly reported symptom (85% of cases) followed by fever (68%), runny nose (66%) and sore throat (62%). There was no statistically significant difference in the percentage of cases with reported symptoms when stratified by age groups of less than school age (<5 years), primary school age (5–11 years), secondary school age (12–17 years) and adults (≥18 years). However, when comparing primary and secondary school-aged children, a significantly higher percentage of primary school children reported fever (76% to 64%; p = 0.02).

**Table 1:** Reported symptoms for 520 of first 1,000 confirmed influenza A(H1N1)pdm09 cases with data by age group and order of case notification, Victoria, 2009.

	Age group (years)					Order of case notification			
Symptom	<5	5–11	12–17	≥18	p value	First 100	Next 900	p value	Total (%)
Cough	16 (76)	106 (85)	178 (86)	143 (86)	0.68	58 (78)	385 (86)	0.08	443 (85)
Fever	13 (62)	95 (76)	133 (64)	111 (67)	0.13	49 (66)	303 (68)	0.77	352 (68)
Runny nose	16 (76)	82 (66)	143 (69)	103 (62)	0.42	40 (54)	304 (68)	0.02	344 (66)
Sore throat	9 (43)	72 (58)	140 (67)	99 (60)	0.07	35 (47)	285 (64)	0.007	320 (62)
Fatigue	5 (24)	45 (36)	62 (30)	62 (37)	0.31	10 (14)	164 (37)	<0.001	174 (33)
Vomiting	3 (14)	17 (14)	20 (10)	16 (10)	0.61	4 (5)	52 (12)	0.15	56 (11)
Diarrhoea	1 (5)	13 (10)	13 (6)	14 (8)	0.53	0	41 (9)	0.002	41 (8)
**Total with symptoms reported**	**21**	**125**	**208**	**166**		**74**	**446**		**520**

Twenty-two cases (2%) were reported to have been hospitalised; eight (1%) of the 707 cases in children (aged less than 18 years) were hospitalised. No deaths were reported. Among the hospitalised children, six (75%) had reported risk factors including asthma (two cases) and one case each with diabetes, pulmonary disease, hypertension and muscular dystrophy.

### Epidemiology in Schools

Children of school age (5–17 years) accounted for 668 of the first 1,000 confirmed cases, for whom data on primary or secondary school attended were available for 599 (90%). Data were also available for three students aged 18 years and six teachers, representing 203 schools. Among the remaining 69 school-aged children, school attended was unknown for 63, two were in higher education institutions, two had not started school, one was not at school and the other was an overseas visitor.

One school accounted for 77 confirmed cases and six schools (3%) had between 10 and 25 cases. The remaining schools had less than ten notified cases each, of which most (145 schools, 74%) had two or fewer cases. The school with the largest number of confirmed cases was a selective school with no geographic enrolment restrictions, and the 77 cases’ residences represented 26 of Melbourne’s 30 metropolitan local government areas.

**Figure 2:**
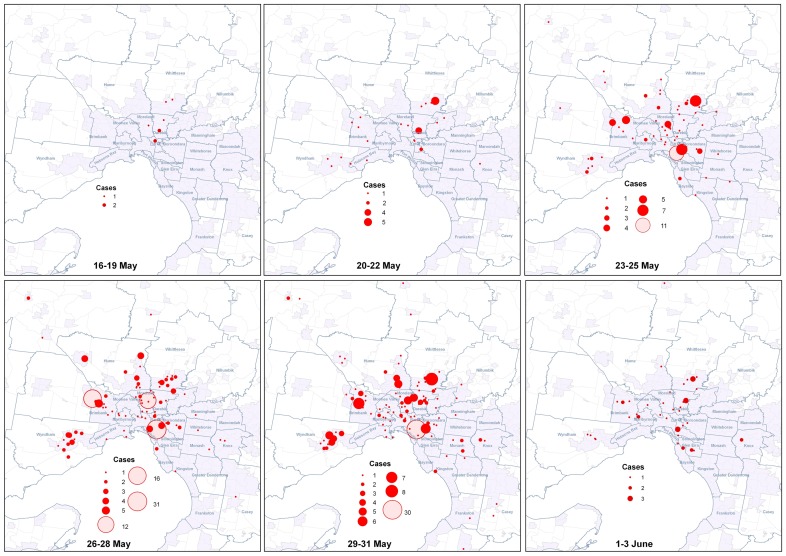
Confirmed influenza A(H1N1)pdm09 cases aged 5–17 years by school and date of onset, Victoria, 2009.

In general, cases appeared first in schools along the northern corridor of the metropolitan area and then became established in outer northern and western suburbs at the same time as a cluster in the inner eastern suburbs (figure 2). Relatively few schools in the eastern suburbs were affected until 3 June. The lower number of cases in the final panel reflects the delay between disease onset and notification, and end of the detailed follow-up of the first 1,000 cases.

**Figure 3:**
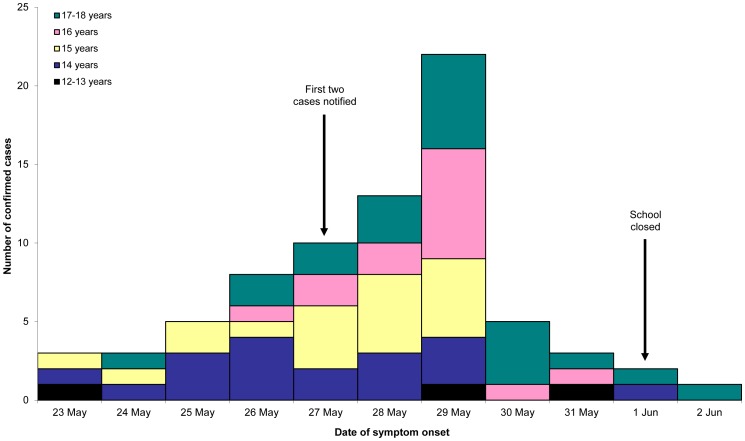
Confirmed influenza A(H1N1)pdm09 cases at School A by date of onset and age group, Victoria, 2009.

An epidemic curve by age group for the school with 77 cases (“School A”) showed a predominance of cases in 14–15 year-olds in the first half of the 11-day period with an increasing proportion of 16–17 year-olds in the second half (figure 3). School A was closed for the week commencing 1 June, nine days after symptom onset in the first case.

**Figure 4:**
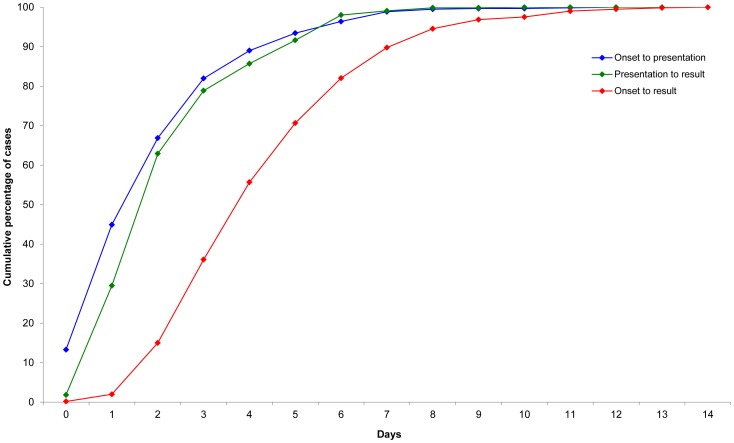
Confirmed influenza A(H1N1)pdm09 cases aged 5–17 years by days from onset to specimen collection and test result, Victoria, 2009.

The median time between illness onset and notification of laboratory confirmation among school children was four days (interquartile range [IQR]: 3–6) (figure 4). The median time from illness onset to medical practitioner presentation and specimen collection was two days (IQR: 1–3), as was specimen collection to confirmatory laboratory result, following which the Department of Health was notified within 12 hours.

### Treatment and Prophylaxis

Treatment data were available for 897 cases (90%) of whom 206 (23%) were prescribed treatment doses of oseltamivir. The proportion of the 691 cases (77%) who did not receive oseltamivir, was significantly higher among school-aged children (80%) compared to adults (73%) and those less than school age (62%) (p = 0.009). Most cases (666/691, 96%) who did not receive oseltamivir were not eligible because more than 48 hours had elapsed since symptom onset. For the remaining 25 cases, the reason was not stated for 14, five were pregnant, alternate treatments were prescribed for four, one declined treatment, it was contraindicated in another and one was unable to source oseltamivir.

Of the 666 cases ineligible for oseltamivir treatment because more than 48 hours had elapsed since symptom onset, 253 (38%) had a specimen collected within one day of symptom onset. Laboratory confirmation was made within one day of specimen collection for 182 (27%) cases and within two days for 417 (63%). Only 69 (10%) of the 666 cases were notified within two days of onset.

Follow-up of cases identified 5,825 eligible contacts to whom oseltamivir prophylaxis doses were distributed. Contacts were not identified for 71 (7%) cases. The number of contacts per case was significantly higher for school-aged children (median = 4, IQR: 3–7) compared to adults (median = 4, IQR: 2–6) (p<0.0001).

### Comparison between the First 100 and Next 900 Cases

Due to the increasing workload associated with the rapid rise in notifications, follow-up of cases was by necessity less complete as the epidemic evolved. We therefore compared the first 100 cases to the following 900 to determine if the different approach to follow-up resulted in any substantial differences in outcome.

Symptoms were reported for 74% of the first 100 cases compared to 50% of the following 900 (p<0.001). However, with the exception of fever which was similar for both groups, specific symptoms were reported for a lower proportion of the first 100 cases (table 1). A non-significantly lower proportion of the next 900 cases were hospitalised (2.1% versus 3.0%) (p = 0.57). No difference between the two groups was observed for the time from onset to specimen collection (p = 0.91) but it took longer for the group of 900 cases to be diagnosed following specimen collection (median = 2 days, IQR: 1–3 versus median = 1 day, IQR: 1–2) (p<0.0001). A significantly higher number of contacts for the first 100 cases (median = 10, IQR: 6–21) were followed up compared to the following 900 (median = 4, IQR: 3–6) (p<0.0001). The median number of contacts per school-aged child was 12 (IQR: 7–31) and nine (IQR: 6–12) for adults who comprised the first 100 cases, but was four (IQR: 3–6) for school-aged children and three (IQR: 2–5) for adults in the group of 900 cases.

## Discussion

Comprising two-thirds of the first 1,000 notified cases, this study is consistent with a review of serological studies that estimated a higher cumulative incidence of influenza A(H1N1)pdm09 infection (prior to the initiation of population-based vaccination against the pandemic strain) in school-aged children of 24–43% compared to pre-school-aged children (16–28%), young adults (12–15%) and older adults (2–3%) [12]. Further evidence of the pivotal role of school-aged children in the spread of influenza A(H1N1)pdm09 was demonstrated in this study by transmission within and from School A, which alone accounted for 8% of the first 1,000 notified cases. The school drew its student population from across the Melbourne metropolitan area, enabling wide geographic dissemination of cases. Rapid transmission had occurred through all the school’s year levels before cases were recognised and student interactions restricted by school closure.

Transmission was also likely facilitated by the generally mild clinical presentation, as evidenced by 32% of notified cases without a reported fever and only 2% being hospitalised, consistent with findings from elsewhere around the globe [13], [14]. Detection of the local epidemic was probably further delayed by the initial case definition criterion for testing of recent overseas travel. Thus, many – presumably infectious – cases were probably not tested, or even saw a clinician, for their illnesses. This hypothesis is supported by modelling which suggested community transmission of the pandemic virus was most likely established in Victoria by late April and was certainly established by the time of its detection [15]. Although the case definition for departmentally authorised testing of suspected influenza A(H1N1)pdm09 cases required a fever, nearly one third of the cases were sampled for influenza testing without a fever. The reason for this is unclear, but given these cases had at least one other reported symptom suggests that other clinical criteria for testing were being recognised by clinicians.

School closure is a commonly suggested mitigation measure for influenza pandemics and the pandemic plans of Australia and Victoria provided for this contingency [8], [16]. Closure of schools to control influenza epidemics and pandemics has been used to varying effect, with timing of the closure(s) – as well as trigger, extent and length – of crucial importance for the intervention’s effectiveness [17]. Modelling using US [18], [19] and Australian [20], [21] populations has suggested school closure can be effective at reducing the final attack rate (cumulative incidence) of influenza but the magnitude of the reduction is highly variable. This variation is likely due to assumptions about differential attack rates in adults and children, the extent of mixing and contact outside school, and the number of symptomatic cases before closure is implemented [21].

In general though, school closure is modelled to be most effective if schools are closed early and remain closed until prevalence returns to low levels and children and teenagers stay at home during closure. There is evidence that closure of kindergartens and schools in Hong Kong for up to one month prior to the commencement of the 2009 summer vacation was effective in the mitigation of influenza A(H1N1)pdm09, with an estimated 70% reduction in intra-age transmission concurrent with school closures [22]. Furthermore, a study in two communities in Dallas/Fort Worth, Texas indicated that reported rates of respiratory illness were lower in a community which closed its schools for eight consecutive days compared to another community in which no schools were closed [23]. However, closure was implemented early when influenza activity was low.

The approach to school closure in Victoria applied to specific schools and classrooms in which two or more confirmed cases had been identified, for the duration of one week. With the exception of isolation for confirmed cases there were no restrictions of student movements. Our study has confirmed the need for a pre-emptive decision on school closure as indicated by theory and practice; in Victoria too few schools were closed too late and for too short a period to have had any discernible impact on the impact of influenza A(H1N1)pdm09 transmission. Specifically in School A the delay between disease onset and notification meant transmission in the school was already well established before the need to close it was identified.

The rapid emergence of affected schools and modelling that estimated establishment of community transmission in Victoria around late April [11], [15] suggested influenza A(H1N1)pdm09 prevalence was high by the time it was detected, and probably too late for widespread school closure to be effective. Whilst pre-emptive, widespread and extended school closure is anticipated to effectively interrupt the transmission of pandemic influenza, it raises concerns about expected compliance with social restrictions, workforce shortages and economic impacts. A study of Victorian households affected by school and classroom closures found 90% of households understood what they were meant to do in the quarantine period [24] and 85% complied with the requirement to stay at home [25]. However, these households were only affected by closures of up to one week and this contrasts with a study among families in Western Australia, which found that school closures caused considerable disruption for families in arranging childcare and poor compliance among those placed in home quarantine [26].

Whilst more than 6,000 treatment and prophylactic doses of oseltamivir associated with the first 1,000 notified cases were distributed to cases and contacts, antiviral treatment could rarely be delivered to cases or their close contacts within 48 hours of symptom onset. It is likely that much of this distribution inefficiency was a consequence of its centralised nature and delays associated with notification. However this centralised system during the *Contain* phase was considered necessary as access to oseltamivir from the National Medical Stockpile was conditional on laboratory confirmation of cases.

Several limitations were associated with the methods of case identification and data collection in this study. The presence of symptoms as a criterion for testing meant that those with subclinical infections were not represented, and although only 52% of first 1,000 cases had recorded symptoms, that a further 39% of cases had a reported illness onset date suggests that most of remainder were missing data. Data quality and the capacity of case investigation officers to follow up cases completely and undertake contact tracing is likely to have progressively diminished as the number of notified cases increased. This suggestion is supported by the difference in reported symptoms and higher median number of contacts followed up per case for the first 100 notified cases compared to the following 900 cases.

Many countries are now reflecting on their 2009 pandemic experiences and responses to review and revise their pandemic plans. Influenza A(H1N1)pdm09 had a generally mild clinical course resulting in apparent widespread dissemination in Victorian school children prior to its detection, meaning that school closure, particularly short-term and isolated closures, were of little or no benefit as a mitigation measure. Pandemic plans need to be refined and flexible to incorporate such scenarios. Indeed, depending on the perceived pandemic severity, it may be better to keep schools open and waive the requirement for laboratory confirmation earlier and to treat clinically compatible children cases, or recommend nothing more than standard respiratory precautions for those exhibiting symptoms. Furthermore, in the wake of this experience consideration should be given to a decentralised, or direct clinician access to the Australia’s National Medical Stockpile, model of antiviral distribution during the early phases of a pandemic. Certainly it is important to include the ramifications of observations from this study in revised pandemic plans.
